# Identification of antibiotic pairs that evade concurrent resistance via a retrospective analysis of antimicrobial susceptibility test results

**DOI:** 10.1016/s2666-5247(21)00118-x

**Published:** 2021-07-23

**Authors:** Andrew M Beckley, Erik S Wright

**Affiliations:** Department of Biomedical Informatics, University of Pittsburgh, Pittsburgh, PA, USA; Department of Biomedical Informatics, University of Pittsburgh, Pittsburgh, PA, USA; Pittsburgh Center for Evolutionary Biology and Medicine, Pittsburgh, PA, USA

## Abstract

**Background:**

Some antibiotic pairs display a property known as collateral sensitivity in which the evolution of resistance to one antibiotic increases sensitivity to the other. Alternating between collaterally sensitive antibiotics has been proposed as a sustainable solution to the problem of antibiotic resistance. We aimed to identify antibiotic pairs that could be considered for treatment strategies based on alternating antibiotics.

**Methods:**

We did a retrospective analysis of 448 563 antimicrobial susceptibility test results acquired over a 4-year period (Jan 1, 2015, to Dec 31, 2018) from 23 hospitals in the University of Pittsburgh Medical Center (Pittsburgh, PA, USA) hospital system. We used a score based on mutual information to identify pairs of antibiotics displaying disjoint resistance, wherein resistance to one antibiotic is commonly associated with susceptibility to the other and vice versa. We applied this approach to the six most frequently isolated bacterial pathogens (*Escherichia coli, Staphylococcus aureus, Klebsiella pneumoniae, Enterococcus faecalis, Pseudomonas aeruginosa*, and *Proteus mirabilis*) and subpopulations of each created by conditioning on resistance to individual antibiotics. To identify higher-order antibiotic interactions, we predicted rates of multidrug resistance for triplets of antibiotics using Markov random fields and compared these to the observed rates.

**Findings:**

We identified 69 antibiotic pairs displaying varying degrees of disjoint resistance for subpopulations of the six bacterial species. However, disjoint resistance was rarely conserved at the species level, with only 6 (0·7%) of 875 antibiotic pairs showing evidence of disjoint resistance. Instead, more than half of antibiotic pairs (465 [53·1%] of 875) exhibited signatures of concurrent resistance, whereby resistance to one antibiotic is associated with resistance to another. We found concurrent resistance to extend to more than two antibiotics, with observed rates of resistance to three antibiotics being higher than predicted from pairwise information alone.

**Interpretation:**

The high frequency of concurrent resistance shows that bacteria have means of counteracting multiple antibiotics at a time. The almost complete absence of disjoint resistance at the species level implies that treatment strategies based on alternating between antibiotics might require subspecies level pathogen identification and be limited to a few antibiotic pairings.

**Funding:**

US National Institutes of Health.

## Introduction

Modern health care is not only reliant on antibiotics to treat infectious disease but also to prevent infections during surgery or immune suppression. Escalating levels of antibiotic resistance impose a substantial burden on medical practice. It is generally believed the evolution of antibiotic resistance is an inevitable consequence of antibiotic use. Antibiotic resistance can arise through de-novo mutation or acquisition of resistance alleles.^1^ The spread of resistance is exacerbated by the fact that the acquisition of resistance to one antibiotic might result in cross-resistance to other antibiotics. However, in some cases, evolution of resistance to one antibiotic confers increased sensitivity to another in a phenomenon known as collateral sensitivity. Alternating between antibiotic pairs that induce collateral sensitivity has been proposed as a sustainable solution to the problem of antibiotic resistance.^2,3^ Yet, treatment strategies based on alternating between antibiotics have generally failed to mitigate the rise of resistance.^4–6^ One potential explanation is that the tested cycling intervals are longer than a hospital stay, preventing a single bacterial population from becoming trapped in an evolutionary trade-off. Another possibility is that clinical trials have used antibiotic pairs that do not offer a sufficient degree of collateral sensitivity. Therefore, additional clinical knowledge about appropriate antibiotic pairings could help avert resistance progression among pathogens.

Experimental evolution has been used extensively to explore the collateral effects of adaptation to antibiotics.^7^ Antibiotic pairs exhibiting collateral sensitivity have been identified in vitro for *Escherichia coli*,^8,9^ *Enterococcus faecalis*^10^, *Pseudomonas aeruginosa*,^11–13^ and *Staphylococcus aureus.*^14^ Similarly, collaterally sensitive drug pairs have been proposed for the treatment of some cancers based on the results of in-vitro evolution experiments.^15^ Drug pairs displaying reciprocal collateral sensitivity (ie, in both directions) are particularly promising because of the possibility that they could be alternated indefinitely without multidrug resistance arising. In a fraction of cases, the mechanism underlying collaterally sensitive interactions has been identified.^16^ For example, aminoglycosides rely on membrane potential for cellular uptake, and resistance can develop via a reduction of the proton motive force. This reduction of the proton motive force will, in turn, decrease the efficacy of efflux pumps required for the expulsion of other antibiotics, thus increasing sensitivity.^17^

There are some practical barriers to implementing treatment strategies on the basis of collateral sensitivity.^3^ First, collaterally sensitive antibiotic pairs can differ among strains of the same species,^18,19^ making it more difficult to identify generalisable treatment strategies applicable to a wide variety of pathogenic strains. Second, different resistance mutations can lead to either cross-resistance or collateral sensitivity,^13,20^ decreasing the dependability of collateral sensitivity as a treatment strategy. Cross-resistance presents a potential route for pathogens to escape the desired effects of alternating between antibiotics. Third, a pathogen’s previous exposure to antibiotics is generally unknown because it is often difficult to track the movement of individual strains between patients. For this reason, chronic infections with the same pathogen might achieve the most success with treatments based on collateral sensitivity.^12^ Fourth, the per-generation rate at which a pathogen reverts to sensitivity is often much lower than the rate at which it develops resistance.^8–10,12,13^ These rate differences could prevent pathogens from becoming susceptible (ie, crossing the clinical breakpoint) during the course of treatment with another antibiotic. Finally, the order of drug application might matter in some circumstances because evolution is not a commutative process.^11,21^

Studies supporting collateral sensitivity as a viable strategy have been largely based on in-vitro evolution experiments. It is well known that adaptations in vitro often differ from those in vivo because of the difference in growth conditions.^22,23^ Within an infection, pathogens are exposed to a different growth substrate (ie, food) and antibiotic dosage profile than in vitro, while contending with other microorganisms and the immune system. Additionally, the traditional definition of collateral sensitivity as a decrease in minimum inhibitory concentration is different from the clinical definitions of susceptibility and resistance. We sought to circumvent these challenges by identifying antibiotic pairs that display disjoint resistance, in which resistance to one antibiotic in the clinic is associated with susceptibility to another and vice versa. These antibiotic pairs serve as promising candidates for treatment strategies based on alternating between antibiotics. In this study, we present a retrospective analysis of antimicrobial susceptibility test results collected over 4 years. We used a scoring metric based on mutual information to identify pairs of antibiotics displaying disjoint resistance among six clinically relevant pathogens. We then extended our analysis from pairs to triplets of antibiotics, seeking sets of antibiotics where triplet resistance was rarer than expected from knowledge of the pairs alone.

## Methods

### Dataset of antimicrobial susceptibility test results

We retrieved all available antimicrobial susceptibility test results done for the University of Pittsburgh Medical Center (UPMC) across a 4-year period (2015–18), totalling 448 563 susceptibility test results. UPMC is a hospital system located in northeastern USA admitting 382 000 inpatients per year. Our dataset consists of inpatient and outpatient test results assigned to susceptible, intermediate, or resistant in accordance with Clinical Laboratory Standards Institute (CLSI) protocols. Susceptibility testing for each hospital is done by either an in-house clinical microbiology laboratory or an accredited third-party clinical laboratory service. All submitting laboratories use quality control strains as recommended by CLSI, and all UPMC laboratories carry a Clinical Laboratory Improvement Amendments certification and are accredited by the College of American Pathologists. The data was extracted from the laboratory information management system and deidentified via an honest broker (Health Record Research Request service, University of Pittsburgh Office of Research, Health Sciences). For each patient isolate, the dataset contains the result date, organism identification, and antimicrobial susceptibility test results. All susceptibility results were included, regardless of patient demographics, comorbidities, or length of stay. Antibiotic abbreviations and classes are included in appendix 1 (pp 2–3). This study was approved by the University of Pittsburgh (Pittsburgh, PA, USA) institutional review board (IRB PRO17110307).

### Identification of rare resistances

In this analysis, we aimed to both identify antibiotic-pathogen pairs where resistance is rarely observed and to exclude these pairs from the disjoint resistance analysis. To identify rare resistances, we narrowed the entire dataset to the first isolate per patient per year to minimise the likelihood of redundant isolates. Organisms that comprised at least 1% of the reduced dataset were included in the analysis of rare resistances. For this analysis, we only included the antibiotics that were tested at least 1000 times per year against a species to focus on the most frequently tested antibiotics for each pathogen. We then reported the subset of antibiotics where resistance was rare, defined as a resistance rate less than 1%.

### Mutual information score

We developed a score on the basis of mutual information to identify antibiotic pairs displaying concurrent resistance, independence, or disjoint resistance. Mutual information quantifies the degree of dependence between two antibiotic susceptibility test results (X and Y) by measuring the amount of information gained about one test result (X) by knowing that of the other (Y). Because mutual information captures the deviation from independence between the individual antibiotics of a pair, it is well suited for identifying disjoint resistance (appendix 1 pp 4–5). Susceptibility test results for pairs of antibiotics (X/Y) belong to one of four possible states: susceptible/susceptible, susceptible/resistant, resistant/susceptible, or resistant/resistant. The component information (CI) for each state (X/Y) was calculated according to the formula:

$$\text{CI}\left(X/Y\right)=P\left(X/Y\right)\log\left(\frac{P\left(X/Y\right)}{P\left(X\right)P\left(Y\right)}\right)$$

where *P* is probability. The mutual information score (MIS) was then defined as:

MIS=(CI[susceptible/susceptible]+CI[resistant/resistant]−CI[susceptible/resistant]+CI[resistant/susceptible])

Concurrent resistance manifests as an X/Y bias toward susceptible/susceptible and resistant/resistant states, resulting in a positive MIS. Conversely, an X/Y bias toward susceptible/resistant and resistant/susceptible due to disjoint resistance would result in a negative MIS. The MIS is maximised (0·7) when susceptibility to one antibiotic always predicts susceptibility to another antibiotic and similarly for non-susceptibility. The MIS is minimised (−0·7) when resistance to one antibiotic always predicts susceptibility to another and vice versa. For simplicity, we categorised the MISs as independent (0·0 < |MIS| < 0·05), weak (0·05 ≤ |MIS| < 0·1), moderate (0·1 ≤ |MIS| < 0·2), or strong (|MIS|≥ 0·2) based on the absolute value of the average MIS of each antibiotic pair (ie, |MIS|).

We separated the dataset into independent datasets from non-overlapping 2-year periods (2015–16 and 2017–18) containing the first isolate per patient and analysed them separately to determine repeatability. Selecting only the first isolate per patient was used to control for potential resampling of the same isolate. For each of the six most frequently isolated species, we tabulated the number of susceptible/susceptible, susceptible/resistant, resistant/susceptible, and resistant/resistant test results for each pair of antibiotics tested against at least 100 isolates per species in both 2-year periods (appendix 2). We required the resistance rate of each antibiotic for each species to be greater than 1% and less than 99%, so that we have the opportunity to observe many cases of susceptibility and non-susceptibility. Zeros were replaced with a pseudocount of one to prevent undefined results. Rates of intermediate results were low (<4% of all results per species) and were classified as resistant for this analysis.

### Conditional MIS

Although our dataset does not contain strain-level pathogen identification, disjoint resistance could be specific to particular subpopulations of a bacterial species. To identify subpopulations in our non-overlapping 2-year datasets, we conditioned on resistance to each antibiotic included in the species-level analysis. Conditioning on resistance resulted in a set of antimicrobial susceptibility test results for subpopulations composed of all isolates resistant to a given antibiotic. Then, the MIS was calculated for all antibiotic pairs tested together against at least 100 isolates except the conditioning antibiotic used to define each subpopulation.

### Predicting rates of resistance to antibiotic triplets

A Markov random field was used to predict the probability of all eight possible states (ie, susceptible/susceptible/susceptible to resistant/resistant/resistant) for triplets of antibiotics from their pairwise counts (∅_*A/B*_)—ie, the number of susceptible/susceptible, susceptible/resistant, resistant/susceptible, and resistant/resistant. This approach models triplets of antibiotics as a fully-connected undirected graph by which each antibiotic can affect the others. In this manner, triplet states composed of pairs that appeared frequently are also predicted to be more frequent. A partition function (*Z*) is used to normalise the distribution:

$$\text{Z}=\underset{A,B,C\in {\left\{\text{susceptible, resistant}\right\}}^{3}}{\sum }\sqrt{\emptyset \left(\text{A}/\text{B}\right)*\emptyset \left(\text{A}/\text{C}\right)*\emptyset \left(\text{B}/\text{C}\right)}$$

where ∅_*A/B*_, ∅_*A/C*_, and ∅_*B/C*_ are the observed counts of the three corresponding paired results that comprise each of the eight triplet states. For example, in the triplet state of susceptible/resistant/susceptible the value of ∅_*A/B*_ would be the count of ∅_*susceptible/resistant*_, ∅_*A/C*_ would be the count of ∅_*susceptible/susceptible*_, and ∅_*B/C*_ would be the count of ∅_*resistant/susceptible*_. The probability (*p*) of observing a given result can then be calculated as:

$$p\left(A/B/C\right)=\frac{1}{\text{Z}}\sqrt{\emptyset \left(\text{A}/\text{B}\right)*\emptyset \left(\text{A}/\text{C}\right)*\emptyset \left(\text{B}/\text{C}\right)}$$

In this way, the Markov random field accounts for the frequencies of each pairwise state without knowledge of the observed triplet frequencies. For each of the five most prevalent pathogens, resistance rates were predicted for all triplets of antibiotics that were included in the mutual information score analysis and were tested together against at least 100 isolates.

### Statistical analysis

We used Fisher’s Exact Test with a Bonferroni corrected p value threshold of 0·01 to test for independence between antibiotic pairs in the MIS analysis and the conditional MIS analysis. We used Spearman’s Rank Order Correlation test to determine the repeatability of MIS results between the 2015–16 and 2017–18 datasets. We used Welch’s t-test to test for differences in inter-class and intra-class MIS means. We used a one-sided binomial test based on the expected probability of paired class frequency, the number of negative MIS results per class pairing, and the total number of MIS results per class pairing to test for an over-representation of paired antibiotic classes in the conditional MIS analysis. We also used a one-sided binomial test based on the expected probability of each triplet, the number of observed test results, and the total number of tests performed against a given triplet to quantify the likelihood of observing the frequency of each triplet result by chance. Statistical significance was determined by whether a frequency was below a Bonferroni corrected p value threshold of 0·01 for the number of class pairings per species. All analyses were done in R (version 3.6.0).

### Role of the funding source

The funder had no role in study design, data collection, data analysis, data interpretation, or writing of the report.

## Results

**Figure 1:**
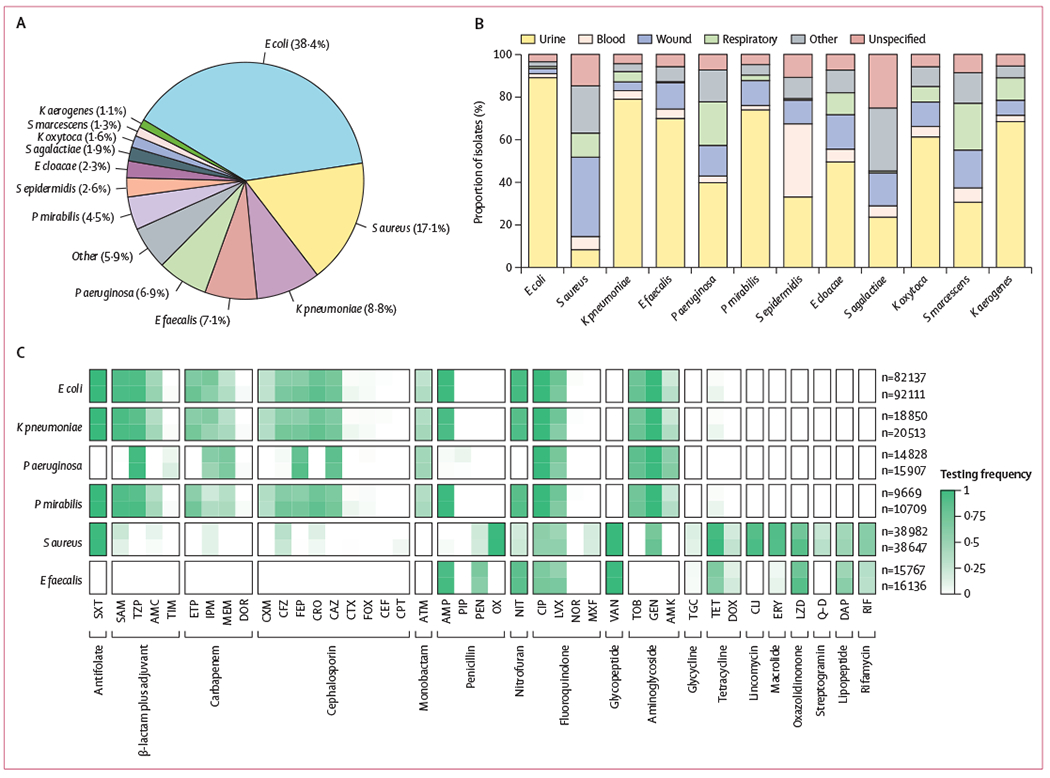
Characteristics of the antimicrobial susceptibility test dataset (A) Species composition of isolates in the dataset (2015–18; total n=448 563). (B) Isolation sources by species. (C) Frequency of testing of different antibiotics (grouped by antibiotic class) by species, in the two 2-year time periods (n=216 095 in 2015–16; n=232 468 in 2017–18). Testing frequency for each species is shown in the top row for 2015–16 and in the bottom row for the 2017–18 dataset. Numbers of isolates of each species in each time period are shown to the right. *E cloacae=Enterobacter cloacae. E coli=Escherichia coli. E faecalis=Enterococcus faecalis. K aerogenes=Klebsiella aerogenes. K oxytoca=Klebsiella oxytoca. K pneumoniae=Klebsiella pneumoniae. P aeruginosa=Pseudomonas aeruginosa. P mirabilis=Proteus mirabilis. S agalactiae=Streptococcus agalactiae. S aureus=Staphylococcus aureus. S epidermidis=Staphylococcus epidermidis. S marcescens=Serratia marcescens.* AMC=amoxicillin-clavulanic acid. AMK=amikacin. AMP=ampicillin. ATM=aztreonam. CAZ=ceftazidime. CEF=cefalotin. CFZ=cefazolin. CIP=ciprofloxacin. CLI=clindamycin. CPT=ceftaroline. CRO=ceftriaxone. CTX=cefotaxime. CXM=cefuroxime. DAP=daptomycin. DOR=doripenem. DOX=doxycycline. ERY=erythromycin. ETP=ertapenem. FEP=cefepime. FOX=cefoxitin. GEN=gentamicin. IPM=imipenem. LVX=levofloxacin. LZD=linezolid. MEM=meropenem. MXF=moxifloxacin. NIT=nitrofurantoin. NOR=norfloxacin. OX=oxacillin. PEN=penicillin. PIP=piperacillin. Q-D=quinupristin–dalfopristin. RIF=rifampicin. SAM=ampicillin-sulbactam. SXT=sulfamethoxazole–trimethoprim. TET=tetracycline. TGC=tigecycline. TIM=ticarcillin-clavulanic acid. TOB=tobramycin. TZP=piperacillin–tazobactam. VAN=vancomycin.

Our dataset contained the results of 448 563 antimicrobial susceptibility test results collected across two non-overlapping 2-year periods (Jan 1, 2015, to Dec 31, 2016, and Jan 1, 2017, to Dec 31, 2018) for 23 hospitals in the UPMC hospital system. The most frequently isolated species were *E coli* (172 139 [38·4%] of 448 563), *S aureus* (76 620 [17·1%] of 448 563), *Klebsiella pneumoniae* (39 363 [8·8%] of 448 563), *E faecalis* (31 903 [7·1%] of 448 563), *P aeruginosa* (30 735 [6·9%] of 448 563), and *Proteus mirabilis* (20 378 [4·5%] of 448 563; figure 1A). The majority (263 945 [58·8%]) of 448 563 isolates originated from urine, but other isolation sources were common for some pathogens (figure 1B).

**Figure 2:**
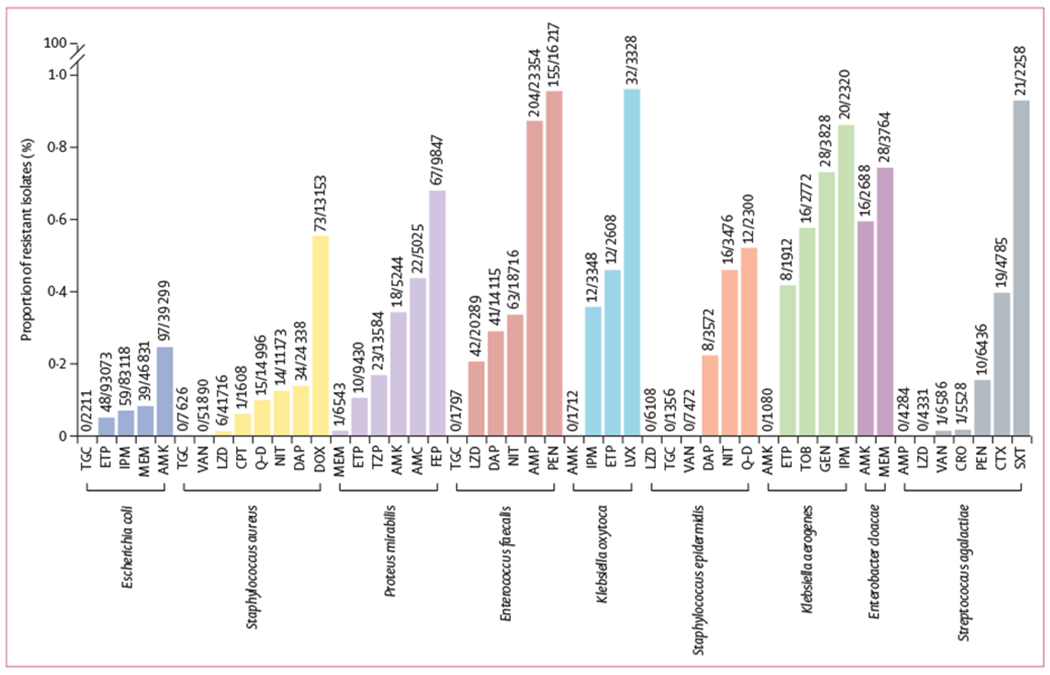
Rarely observed antibiotic resistances by species Rates of resistance (<1%) are shown for antibiotics tested at least 1000 times per first patient isolate during a 4-year period (2015–18). Data above each bar are n/N (ie, number of resistant isolates divided by the total number of isolates tested). AMC=amoxicillin–clavulanic acid. AMK=amikacin. AMP=ampicillin. CPT=ceftaroline. CRO=ceftriaxone. CTX=cefotaxime. DAP=daptomycin. DOX=doxycycline. ETP=ertapenem. FEP=cefepime. GEN=gentamicin. IPM=imipenem. LVX=levofloxacin. LZD=linezolid. MEM=meropenem. NIT=nitrofurantoin. pEN=Penicillin. Q-D=quinupristin–dalfopristin. SXT=sulfamethoxazole–trimethoprim. TGC=tigecycline. TOB=tobramycin. TZP=piperacillin–tazobactam. VAN=vancomycin.

Claims of resistance-proof antibiotics have received considerable criticism in the literature because it is postulated that pathogens can evolve resistance to any antibiotic.^24^ We calculated the set of antibiotic–pathogen pairs that were rarely (<1%) resistant in our dataset (figure 2). There were 12 pathogens collected from 2015–18 meeting our inclusion criteria for this analysis, totalling 309 849 individual isolates of *E coli, S aureus, K pneumoniae, E faecalis, P aeruginosa, P mirabilis, Staphylococcus epidermidis, Enterobacter cloacae, Staphylococcus agalcactiae, Klebsiella oxytoca, Serratia marcescens*, and *Klebsiella aerogenes*. Tigecycline resistance was never observed among *E coli, E faecalis, S aureus*, and *S epidermidis* isolates. Plasmid-mediated tigecycline resistance emerged as recently as 2019 among some clinical pathogens, and rates of tigecycline resistance vary around the world.^25^ Additionally, we did not observe isolates of *S aureus* that were resistant to vancomycin. The US Centers for Disease Control and Prevention has documented only 14 cases of vancomycin resistant *S aureus* in the USA.^26^ Similarly, no ampicillin resistant isolates of *S agalactiae* (group B streptococci) were observed, although isolates with reduced susceptibility have been previously reported.^27^ We did not detect some well-known cases of ubiquitous susceptibility, such as the absence of penicillin resistance among group A streptococci^28^ because they did not reach the threshold of 1000 tested isolates required for inclusion in our analysis.

We observed low, but non-zero, rates of resistance to some antibiotics of last resort. Linezolid resistance was never observed among *S epidermidis* and *S agalactiae*, although we observed six linezolid resistant isolates of *S aureus* (6 [<0·1%] of 41 716). Similarly, rates of carbapenem resistance among Enterobacteriaceae were extremely low for *E coli* and *K pneumoniae. P aeruginosa* was conspicuously absent from figure 2, which reflects a general scarcity of universal treatment options for this pathogen. Overall, 50 (23·1%) of 216 antibiotics tested against at least 1000 isolates per species had resistance rates of less than 1%.

**Figure 3:**
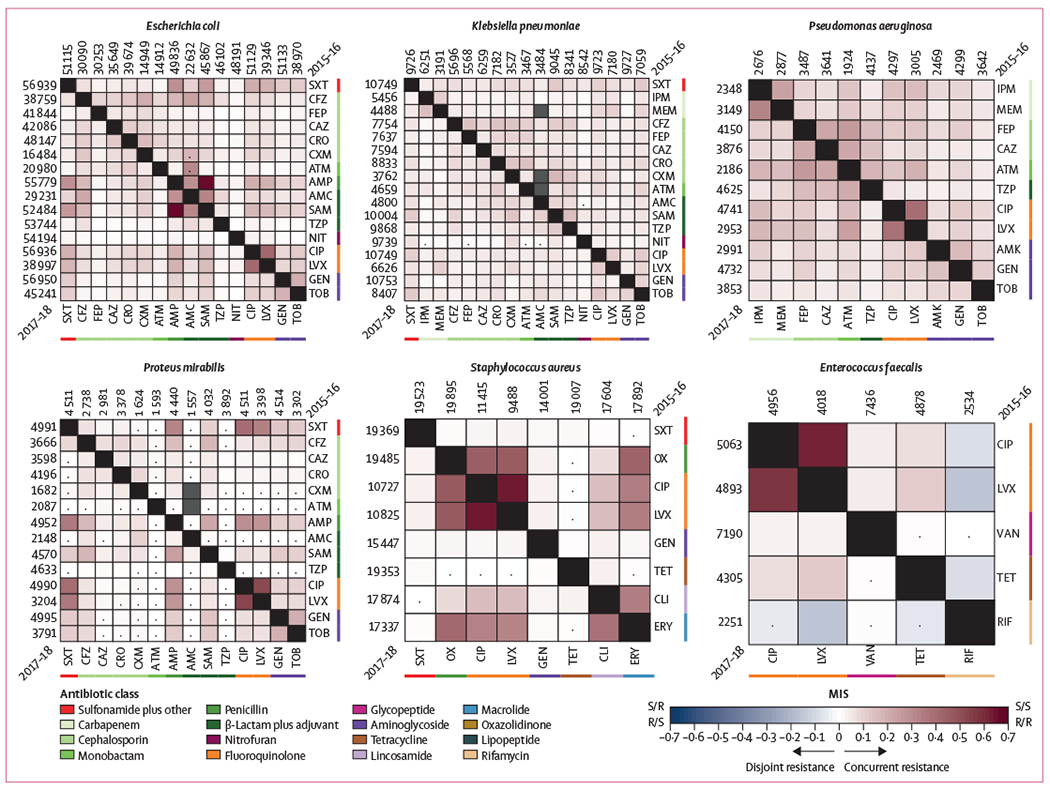
Heatmaps for six species showing the MISs for pairs of antibiotics in 2015–16 and 2017–18 2015–16 is shown in the upper right of each heatmap and 2017–18 in the lower left; the numbers along each side denote the total number of times a drug was tested. Negative MISs (blue) correspond to disjoint resistance, positive MISs (red) correspond to concurrent resistance, and MISs near zero (white) imply independence. Only three cases of statistically significant negative MISs were observed. Antibiotic classes often displayed intra-class concurrent resistance, although many positive MISs were observed between antibiotics belonging to different classes. Gray boxes designate pairs that were tested together less than 100 times, and dots signify no statistical significance (Fisher Exact test, Bonferroni corrected p≥0.01). MIS=mutual information score. AMC=amoxicillin–clavulanic acid. AMK=amikacin. AMP=ampicillin. ATM=aztreonam. CAZ=ceftazidime. CFZ=cefazolin. CIP=ciprofloxacin. CLI=clindamycin. CRO=ceftriaxone. CXM=cefuroxime. ERY=erythromycin. FEP=cefepime. GEN=gentamicin. IPM=imipenem. LVX=levofloxacin. MEM=meropenem. NIT=nitrofurantoin. OX=oxacillin. RIF=rifampicin. SAM=ampicillin–sulbactam. SXT=sulfamethoxazole-trimethoprim. TET=tetracycline. TOB=tobramycin. TZP=piperacillin-tazobactam. VAN=vancomycin.

Antibiotic pairs exhibiting disjoint resistance would trap pathogens in either susceptible/resistant or resistant/susceptible states by preventing them from maintaining the resistant/resistant state. Therefore, we used the MIS of antibiotic pairs to identify pairings whereby the susceptible/resistant and resistant/susceptible results are observed more commonly than expected if resistance to each antibiotic was independent. We calculated the MISs for 875 pathogen–antibiotic pairs across six species in two non-overlapping datasets meeting our inclusion criteria: 240 pairs in *E coli*, 269 pairs in *K pneumoniae*, 110 pairs in *P aeruginosa*, 180 pairs in *P mirabilis*, 56 pairs in *S aureus*, and 20 pairs in *E faecalis*. MISs were repeatable between independent datasets arising from non-overlapping 2-year periods (2015–16 *vs* 2017–18; Spearman’s ρ≥0·84 per pathogen), with rare outliers attributable to changes in testing frequency (figure 1C). An abundance of concurrent resistance was seen across all species (471 [53·8%] of 875 pairs that have MIS>0·05), with *P aeruginosa* having both the greatest proportion (96 [87·3%] of 110 pairs) of positive MISs and most being classified as moderate or strong across both non-overlapping 2-year periods (22 [40·0%] of 55 pairs; figure 3). *K pneumoniae* showed the weakest evidence of concurrent resistance with 116 (85·3%) of 136 pairs being classified as independent or weak.

Strong evidence of concurrent resistance was frequently identified for antibiotics belonging to the same class, because the intra-class average MIS was twice the inter-class average (0·15 *vs* 0·08, Welch’s *t* test p<0·0001). Ciprofloxacin and levofloxacin showed strong concurrent resistance in all species except *K pneumoniae*, and combinations of β-lactam antibiotics also showed strong evidence of concurrent resistance in *E coli*, *P aeruginosa*, and *P mirabilis*.

We observed an almost complete absence of negative MISs (disjoint resistance) among antibiotic pairs (6 [0·7%] of 875 pairs have a MIS<−0·05). The two lowest MISs were for rifampicin paired with levofloxacin (−0·16) and ciprofloxacin (−0·08) among *E faecalis* isolates. Similarly, rifampicin and tetracycline had a negative MIS (−0·08). None of these antibiotic pairings showed reciprocal collateral sensitivity during in-vitro evolution experiments.^10^

**Figure 4:**
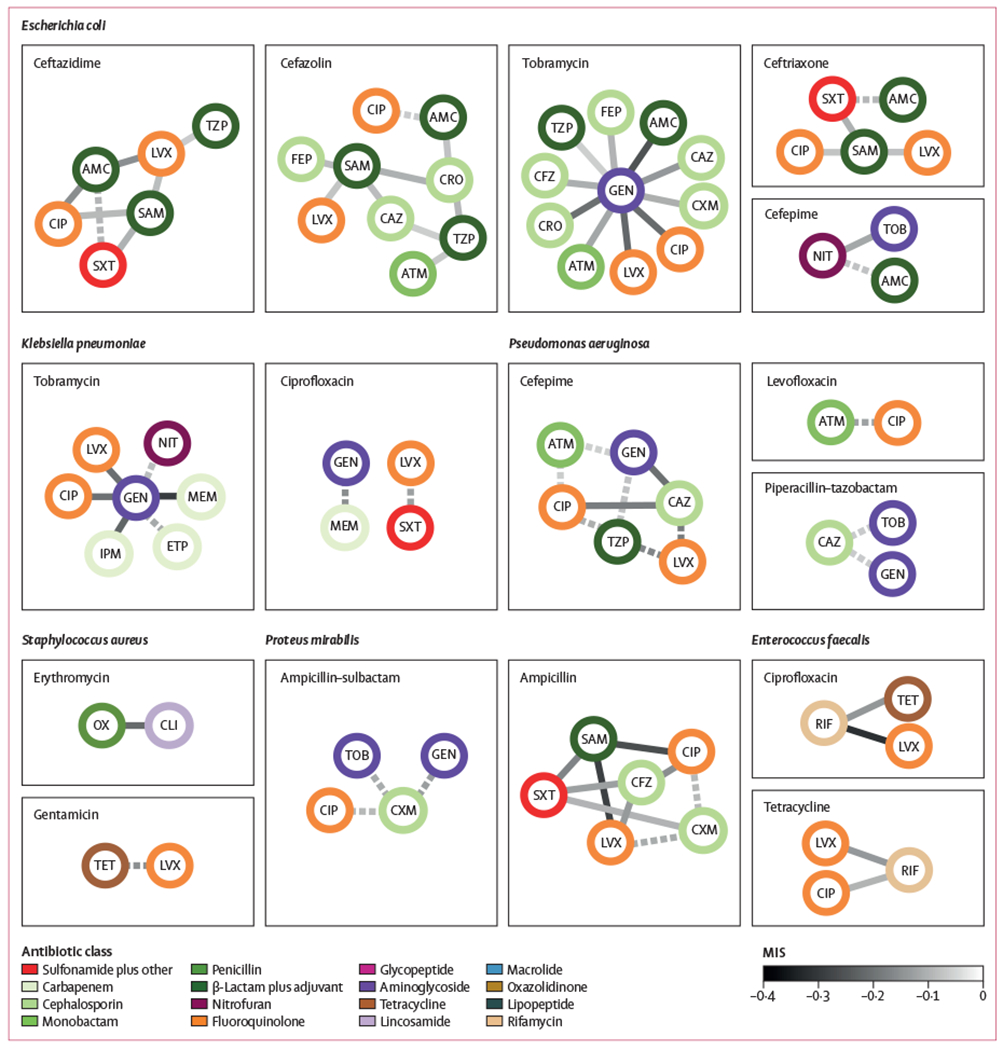
Network display of antibiotic pairs with negative MISs identified in the subset of isolates from each species that were resistant to a given antibiotic Antibiotic pairs are denoted by connected nodes and negative MISs are denoted by edges (ie, connecting lines). Conditioning on resistance to an antibiotic (titles) revealed disjoint resistances that were undiscovered in the analysis of the entire species (figure 3). Antibiotic pairs are shown with MISs of less than −0.05 in both 2015–16 and 2017–18, with dotted lines indicating statistical significance in only one of the 2-year periods and solid lines indicating statistical significance in both of the 2-year periods (Fisher’s Exact test, Bonferroni corrected p<0.01). Nodes are coloured by antibiotic class. MIS=mutual information score. AMC=amoxicillin–clavulanic acid. ATM=aztreonam. CFZ=cefazolin. FEP=cefepime. CAZ=ceftazidime. CIP=ciprofloxacin. CLI=clindamycin. CRO=ceftriaxone. CXM=cefuroxime. ETP=ertapenem. GEN=gentamicin. IPM=imipenem. LVX=levofloxacin. MEM=meropenem. NIT=nitrofurantoin. OX=oxacillin. SAM=ampicillin–sulbactam. SXT=sulfamethoxazole–trimethoprim. TET=tetracycline. TOB=tobramycin. TZP=piperacillin–tazobactam.

It is known that collateral sensitivities are sometimes strain specific within the same species,^19^ which could be masked when only examining species as a single population. We hypothesised that subpopulation-specific disjoint resistance could be exposed by conditioning on resistance to a given drug, which would act as a proxy for subspecies-level classification. This approach revealed 69 cases of disjoint resistance with a MIS less than −0·05 (figure 4). Among these, we observed a statistically significant over-representation of fluoroquinolones paired with β-lactams plus adjuvants tested against *E coli* (one-sided binomial test, Bonferroni corrected p=0·0002) and aminoglycosides paired with carbapenems tested against *K pneumoniae* (p=0·0018). Of the 69 pairs, 12 showed strong disjoint resistance (MIS less than −0·2) with eight of these having an aminoglycoside as one of the antibiotics. Nevertheless, negative MISs were still rare considering the number of possible antibiotic pairings and far greater proportion of positive MISs (1961 pairs have MIS>0·05 at the subpopulation-level).

**Figure 5:**
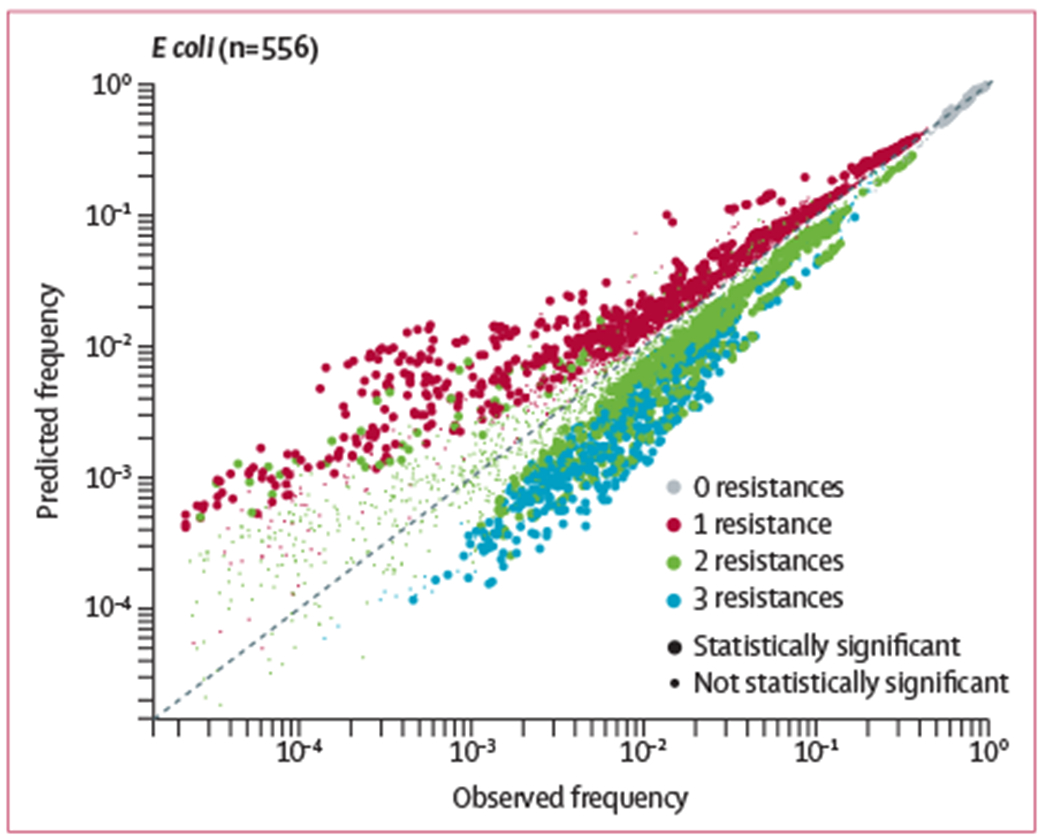
Predicted resistance rates for triplets of antibiotics in *Escherichia coli* A Markov random field was used to predict resistance rates for triplets of antibiotics based on knowledge of the pairs alone. Each point represents one of eight possible susceptibility results (0–3 resistances) for three antibiotics (n=556 triplet combinations), with smaller points denoting no statistical significance (one-sided binomial test, Bonferroni corrected p≥0.01) and larger points denoting statistical significance (p<0.01). Points above the diagonal line indicate overpredicted resistance rates, points below the line indicate underpredicted rates. Data for four other pathogens are shown in appendix 1 (p 7). All results are shown for 2015–16.

It is possible that more than two antibiotics are required to complete a cycle of collateral sensitivity—eg, triplets of antibiotics were previously identified that confer collateral sensitivity in vitro.^9^ These antibiotic combinations would ideally result in lower rates of triplet resistance than predicted based on knowledge of the pairs alone, because cycles of collateral sensitivity might prevent the pathogen from reaching a resistant/resistant/resistant state. We used a Markov random field to estimate rates of observing zero (susceptible/susceptible/susceptible) to three resistances (resistant/resistant/resistant) for triplets of antibiotics based on frequencies of pairwise resistance. This method accurately predicted resistance frequencies for most triplet results (figure 5 and appendix 1 p 7). However, it systematically predicted higher frequencies of triplets containing one resistance (susceptible/susceptible/resistant, susceptible/resistant/susceptible, or resistant/susceptible/susceptible; red points in figure 5 and appendix 1 p 7) and lower frequencies of triplets with three resistances (resistant/resistant/resistant; blue points in figure 5 and appendix 1 p 7) than were observed. Remarkably, we did not detect any exceptions to this rule, that is, statistically significant resistant/resistant/resistant points above the identity line. This finding indicates a complete absence of antibiotic triplets that confer disjoint resistance beyond that observed among pairs.

## Discussion

In this study, we used a large dataset of antimicrobial susceptibility test results to identify promising antibiotic pairs for treatment strategies based on mixing or cycling antibiotics. Unfortunately, we mainly saw concurrent resistance at the species level among antibiotics used to treat six common bacterial pathogens, which corroborates and extends upon previous findings of concurrent resistance between antibiotics.^29^ These concurrent resistance associations could be used to design treatment strategies attempting to avoid cross-resistance between prescribed antibiotics. We also found that triplet resistance occurs more frequently than predicted from the pairs alone, suggesting mechanisms of resistance tend to confer more resistance than expected from pairwise interactions. However, an analysis of subpopulations yielded encouraging results, whereby we identified 69 antibiotic pairs exhibiting disjoint resistance.

Taken as a whole, our results suggest that the rarity of antibiotic pairings exhibiting disjoint resistance poses an additional challenge to successful implementation of empiric treatment strategies on the basis of alternating antibiotics and might partly explain the shortcomings of previous clinical trials testing antibiotic cycling.^4,6,30^ Nevertheless, the subpopulation-specific disjoint resistances that we identified might maintain the hope that strategies based on alternating between antibiotics could be clinically useful. Promisingly, an over-representation of disjoint resistances between fluoroquinolones and β-lactams plus adjuvants in the *E coli* subpopulation analysis aligns with the results of a cycling study, in which periods of increased fluoroquinolone use were associated with decreased resistance rates to amoxicillin–clavulanic acid among extended spectrum β-lactamase-producing isolates.^31^ Additionally, 8 (66.7%) of the 12 strong subpopulation-specific disjoint resistance pairs included an aminoglycoside, a class of antibiotics which are known to cause collateral sensitivity with other classes of antibiotics.^17^

Use of antimicrobial susceptibility tests to detect disjoint resistance has some limitations. First, data are collected from a variety of hospitals, patients, and testing centres that might impose variability in the way that tests are conducted. Second, resampling of the same isolate across multiple patients (eg, outbreaks or transmission chains) might result in biased resistance profiles. These resampling biases would be expected to exaggerate both concurrent and disjoint resistances, even though we rarely observed disjoint resistances. Microbial genomic data could provide a means of mitigating resampling bias but are rarely collected at the hospital scale. Third, our approach is unable to detect disjoint resistance with antibiotics where resistance rates are very low (figure 2) because there are too few cases of resistance observed. Fourth, non-uniform prescribing practices make it infeasible to determine which antibiotics are applied as selective pressure to the pathogens within our dataset. This practice might prevent us from detecting possible cases of disjoint resistance between infrequently prescribed antibiotics. Fifth, disjoint resistance requires reciprocal collateral sensitivity. Unidirectional collateral sensitivity is a weaker requirement that might be more prevalent and sufficient for some treatments. Sixth, our method ignores changes in sensitivity that occur within the same susceptibility designation (ie, susceptible or resistant), such as from sensitivity to hypersensitivity. Hence, we only expect to identify antibiotic pairings in which the minimum inhibitory concentration regularly crosses the breakpoint, although these are arguably the most clinically relevant. Notwithstanding these limitations, there was far more evidence for concurrent than disjoint resistance.

Our initial aim was to identify cases of disjoint resistance in a manner that was most representative of clinical pathogens in their natural environment. The ubiquity of concurrent resistance suggests that evolutionary paths toward multidrug resistance are available for most antibiotic pairings, which would be expected if some resistance mechanisms confer resistance to multiple antibiotics. This conclusion is supported by the fact that pairwise resistance leads to even more resistance than expected when three antibiotics are considered. The only exception appears to be for subpopulations of species whereby evolution might have led to a genotype that exhibits disjoint resistance that is not conserved across all members of the species. This finding poses a somewhat greater challenge for successful implementation of treatment strategies based on alternating antibiotics because isolates must be identified at a subspecies level. Such a strategy could become more viable as pathogen whole-genome sequencing and rapid antibiotic susceptibility tests are more widely adopted. Therefore, we maintain some hope for antibiotic cycling but doubt that it will offer a sustainable and broadly applicable cure for the rise of antibiotic resistance.

## Supplementary Material

1

2

## Research in context

### Evidence before this study

We searched the PubMed database for clinical trial publications before Nov 9, 2020, using the search terms “antibiotic cycling” and “antibiotic mixing” with no language restrictions. Combining these search results with selected reference lists from related studies, we identified six clinical trials comparing resistance rates before and after antibiotic cycling or mixing approaches were implemented, or both. Overall, the evidence supporting cycling or mixing strategies was inconclusive.

One trial found that implementation of a cycling protocol increased antibiotic susceptibility among pseudomonal isolates, as resistance rates to ceftazidime and piperacillin or tazobactam fell during the cycling period. The other five studies concluded there was no effect of cycling or mixing antibiotics on resistance rates. Notably, none of the clinical trials cited in-vitro evidence or otherwise justified their choice of cycled antibiotics.

### Added value of this study

Here we describe how antibiotic pairs that hold promise for treatment strategies based on alternating between antibiotics will display the property of disjoint (ie, mutually exclusive) resistance in the clinic. Using routinely collected clinical microbiology data, we sought pairs of antibiotics displaying disjoint resistance that could be evaluated in future clinical trials. We show that among six common bacterial pathogens, most antibiotic pairs display the opposite property of concurrent resistance. However, disjoint resistance was identified within subpopulations of the six bacterial pathogens. Aminoglycosides were frequent among antibiotic pairs with the strongest evidence of disjoint resistance, similar to the results of in-vitro studies of collateral sensitivity. We also showed that concurrent resistance to three antibiotics occurs more frequently than expected from knowledge of the pairs alone.

### Implications of all the available evidence

The unsuccessful previous attempts to reverse resistance by alternating between antibiotics might be due to the rarity of disjoint resistance at the species level. Most of the promising antibiotic pairs identified in this study were subpopulation specific, suggesting that successful treatment strategies will require strain-level specificity. We also showed that resistance begets more resistance, implying that alternating between three antibiotics is unlikely to be more successful than switching between pairs. Clinical studies are needed to evaluate the antibiotic pairs identified here for their potential application in treatment strategies based on cycling or mixing antibiotics.
