# Supplementary material for: Identification of antibiotic pairs that evade concurrent resistance via a retrospective analysis of antimicrobial susceptibility test results
Source: Lancet Microbe. Author manuscript; Available in PMC 2021 Oct 7. (PMC8496867; doi:10.1016/s2666-5247(21)00118-x)
Supplement: 1 [file NIHMS1744133-supplement-1.pdf]

THE LANCET
Microbe

Supplementary appendix

This appendix formed part of the original submission and has been peer reviewed. We post it as supplied by the authors.

Supplement to: Beckley AM, Wright ES. Identification of antibiotic pairs that evade concurrent resistance via a retrospective analysis of antimicrobial susceptibility test results. *Lancet Microbe* 2021; published online July 23. https://doi.org/10.1016/S2666-5247(21)00118-X.

Appendix

Identification of antibiotic pairs that evade concurrent resistance via a retrospective analysis of antimicrobial susceptibility test results

By Andrew M. Beckley & Erik S. Wright

Table of Contents

**Antibiotic abbreviation table**	**2**
**Figure S1**	**4**
**Figure S2**	**6**
**Figure S3**	**7**

Table S1: Antibiotic abbreviations and class designations

Antibiotic Name	Abbreviation	Class
Amikacin	AMK	Aminoglycoside
Amoxicillin-clavulanic acid	AMC	β-lactam + adjuvant
Ampicillin	AMP	Penicillin
Ampicillin-sulbactam	SAM	β-lactam + adjuvant
Aztreonam	ATM	Monobactam
Cefazolin	CFZ	Cephalosporin
Cefepime	FEP	Cephalosporin
Cefotaxime	CTX	Cephalosporin
Cefoxitin	FOX	Cephalosporin
Ceftaroline	CPT	Cephalosporin
Ceftazidime	CAZ	Cephalosporin
Ceftriaxone	CRO	Cephalosporin
Cefuroxime	CXM	Cephalosporin
Cephalothin	CEF	Cephalosporin
Ciprofloxacin	CIP	Fluoroquinolone
Clindamycin	CLI	Lincosamide
Colistin	CST	Polymyxin
Daptomycin	DAP	Lipopeptide
Doripenem	DOR	Carbapenem
Doxycycline	DOX	Tetracycline
Ertapenem	ETP	Carbapenem
Erythromycin	ERY	Macrolide
Fosfomycin	FOF	Phosphonic acid derivative
Gentamicin	GEN	Aminoglycoside
Imipenem	IPM	Carbapenem
Levofloxacin	LVX	Fluoroquinolone

Linezolid	LZD	Oxazolidinones
Meropenem	MEM	Carbapenem
Moxifloxacin	MXF	Fluoroquinolone
Nalidixic acid	NAL	Quinolone
Neomycin	NEO	Aminoglycoside
Nitrofurantoin	NIT	Nitrofuran
Norfloxacin	NOR	Fluoroquinolone
Oxacillin	OX	Penicillin
Penicillin	PEN	Penicillin
Piperacillin	PIP	Penicillin
Piperacillin-tazobactam	TZP	β-lactam + adjuvant
Quinupristin-dalfopristin	Q-D	Streptogramin
Rifampin	RIF	Rifamycin
Tetracycline	TET	Tetracycline
Ticarcillin-clavulanic acid	TIM	β-lactam + adjuvant
Tigecycline	TGC	Glycycycline
Tobramycin	TOB	Aminoglycoside
Trimethoprim-sulfamethoxazole	SXT	Antifolate
Vancomycin	VAN	Glycopeptide

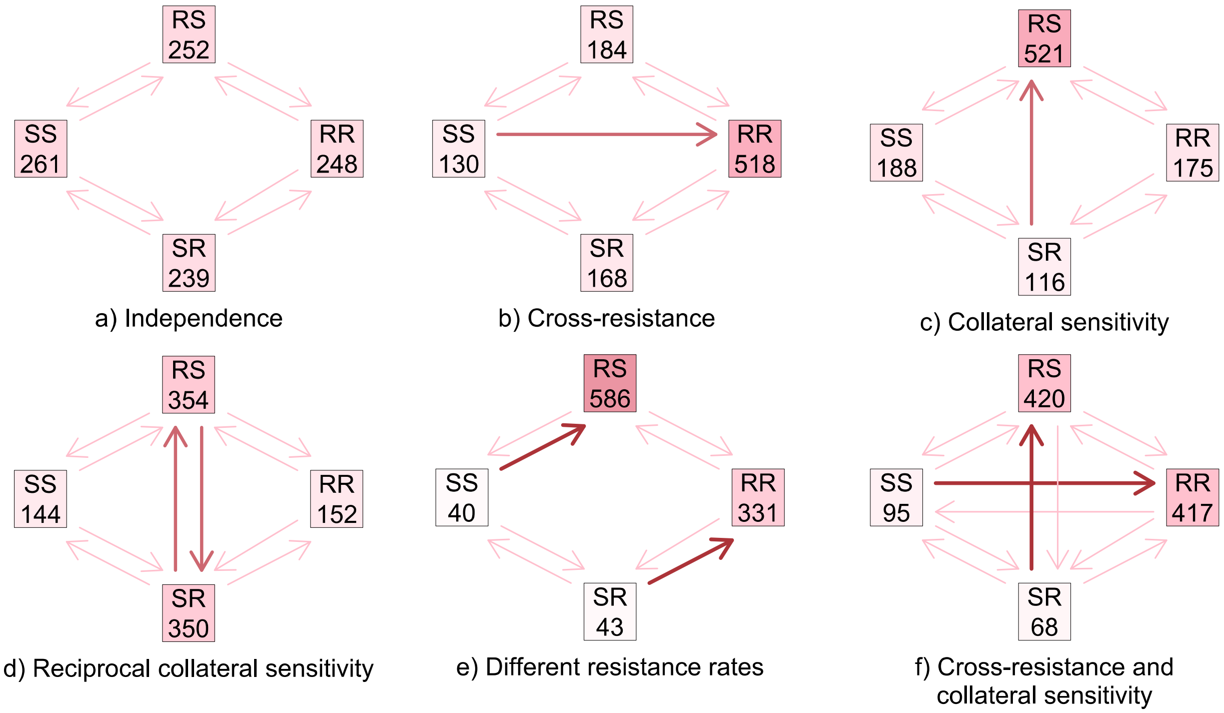

	Mutual Information Score (MIS)	Co-resistance rate	Log-odds ratio	Yule's Y
	$(CI(SS) + CI(RR)) - (CI(SR) + CI(RS))$	$\frac{RR}{SR + RS + RR}$	$\ln \frac{SS * RR}{RS * RR}$	$\frac{\sqrt{SS * RR} - \sqrt{RS * SR}}{\sqrt{SS * RR} + \sqrt{RS * SR}}$
a)	0.018	0.336	0.07	0.02
b)	0.147	0.595	0.78	0.19
c)	-0.111	0.216	-0.61	-0.15
d)	-0.396	0.178	-1.73	-0.41
e)	-0.048	0.345	-0.64	-0.16
f)	0.044	0.461	0.33	0.08

Figure S1. Comparison of scoring methodologies for detecting disjoint resistance. A hidden Markov model was used to simulate the state frequencies of a 1,000 isolate population to compare the efficacy of four different scoring methodologies for identifying disjoint resistance. Isolates are initialized at the state where they are susceptible to both drugs (SS) and move between states with transition probabilities determined by the relative weight of outgoing arrows. Final states after 1,000 transitions

are shown for six different resistance scenarios (a-f). All four scoring methodologies correctly identify independence, cross-resistance, and collateral sensitivity in their respective simulations (a-c). However, the co-resistance rate is unable to distinguish between collateral sensitivity and reciprocal collateral sensitivity (c and d), whereas the MIS, log-odds ratio, and Yule's Y correctly report disjoint resistance under reciprocal collateral sensitivity. The log-odds ratio and Yule's Y incorrectly identify differing resistance rates as collateral sensitivity (c and e), while both the MIS and co-resistance rate correctly identify independence. Finally, all scoring methodologies are blind to cases of matched cross-resistance and collateral sensitivity (f).

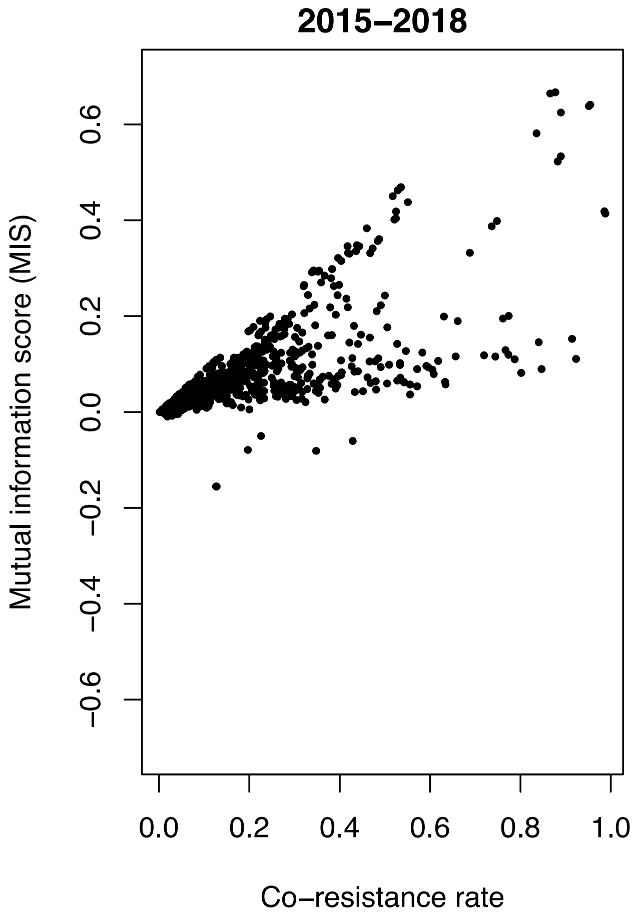

Figure S2. Comparison of MIS to co-resistance rates. High MISs (y-axis) generally had high co-resistance rates (x-axis), however low co-resistance rates do not imply low MISs.

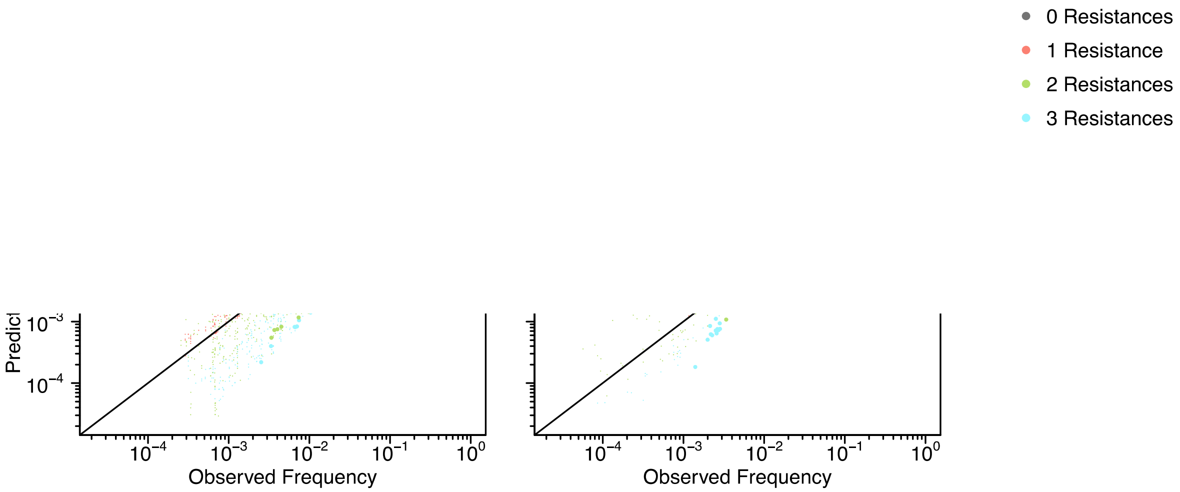

Figure S3. Markov random field predictions. A Markov random field was used to predict triplet resistance frequencies from knowledge of pairwise resistance frequencies alone. Similarly to *E. coli* (Figure 5), resistance rates for triplets containing one resistance were systematically overpredicted while triplets containing three resistance were underpredicted in *K. pneumoniae*, *P. aeruginosa*, *P. mirabilis*, and *S. aureus*. All results are shown for 2015/16. The number (n) of triplet combinations is shown above each plot.
